# Clinical significance of prognostic inflammation-based and/or nutritional markers in patients with stage III gastric cancer

**DOI:** 10.1186/s12885-020-07010-0

**Published:** 2020-06-03

**Authors:** Takahiro Toyokawa, Kazuya Muguruma, Mami Yoshii, Tatsuro Tamura, Katsunobu Sakurai, Naoshi Kubo, Hiroaki Tanaka, Shigeru Lee, Masakazu Yashiro, Masaichi Ohira

**Affiliations:** 1 Department of Gastroenterological Surgery, Osaka City University Graduate School of Medicine, 1-4-3 Asahimachi, Abeno-ku, Osaka, 545-8585 Japan; 2 Department of Gastroenterological Surgery, Osaka City General Hospital, Osaka, Japan

**Keywords:** Gastric cancer, C-reactive protein to albumin ratio, Platelet to lymphocyte ratio, Nutrition, Inflammation

## Abstract

**Background:**

Although many studies have identified several inflammation-based and/or nutritional markers with prognostic value for patients with various types of cancer, the optimal markers and cut-off values for these markers remain obscure. Therefore, this retrospective study aimed to identify optimal markers and their cutoffs.

**Methods:**

We compared prognostic values among established preoperative inflammation-based and/or nutritional markers in 225 patients who underwent R0 resection for stage III gastric cancer. Inflammation-based and/or nutritional markers comprised C-reactive protein to albumin ratio (CAR), neutrophil to lymphocyte ratio (NLR), platelet to lymphocyte ratio (PLR), prognostic nutritional index (PNI), Glasgow prognostic score (GPS), and prognostic index (PI). Time-dependent receiver operating characteristic curves were analyzed to assess predictive ability and to determine the optimal cut-off values. Prognostic factors predicting overall survival (OS) and cancer specific survival (CSS) were analyzed using Cox proportional hazards models.

**Results:**

Multivariate analyses revealed that CAR and PLR cut-off values of 0.47 and 172, respectively, were independent prognostic factors for overall survival (OS) (HR, 2.257; 95% CI, 1.180–4.319; *p* = 0.014 and HR, 1.478; 95% CI, 1.025–2.133; *p* = 0.037, respectively) and cancer-specific survival (CSS) (HR, 2.771; 95% CI, 1.398–5.493; *p* = 0.004 and HR, 1.552; 95% CI, 1.029–2.341; *p* = 0.036, respectively). These results were different from those we previously reported in patients with stage II.

**Conclusions:**

Among inflammation-based and/or nutritional markers, CAR and PLR were independent prognostic factors of OS and CSS in patients with stage III gastric cancer. The optimal markers and their cut-off values should be determined in specific populations.

## Background

Gastric cancer is the second leading cause of cancer-related death worldwide [1]. Advances in diagnosis and treatment modalities have led to favorable outcomes for early-stage gastric cancer, but the postoperative survival of patients with advanced gastric cancer remains poor. Stage III gastric cancer accounts for approximately 15% of gastric cancers, and the reported 5-year overall survival (OS) rate in Japan is 34.8–53.6% [2]. At present, TNM classification is the most generally accepted predictor of long-term outcomes and for selecting adjuvant therapies for gastric cancer in clinical practice. However, clinical outcomes vary even within the same stage because other factors influence outcomes. Therefore, other biomarkers should be identified to predict individual outcomes more precisely and to develop individual treatment strategies for patients with gastric cancer.

Many studies have indicated that not only tumor-related factors, but also patient-related factors such as systemic inflammation and nutritional status, are involved in the prognosis of patients with cancer. Several inflammation-based and/or nutritional markers have recently been developed and preoperative markers, such as C-reactive protein to albumin ratio (CAR) [3, 4], neutrophils to lymphocyte ratio (NLR) [5, 6], platelets to lymphocyte ratio (PLR) [7, 8], as well as prognostic nutritional index (PNI) [9, 10], Glasgow prognostic score (GPS) [11, 12] and prognostic index (PI) [13, 14] are prognostic for various cancers. These markers are promising as clinical prognostic predictors of cancer because they are inexpensive and simple to estimate. On the other hand, the optimal markers and their cut-off value remain debatable and this could cause problems with clinical applications of these markers. We considered that these variations were attributable to different study populations. Therefore, optimal markers and their cut-off values should be determined based on a specific population such as a single tumor stage of each type of cancer to minimize discrepancies. We recently described optimal markers and their cut-off values for patients with stage II gastric cancer [15]. Here, we aimed to determine the prognostic impact of preoperative inflammation-based and nutritional markers in patients with stage III gastric cancer, and to verify whether it differs between stage II and stage III.

## Methods

We retrospectively reviewed clinical data from consecutive patients with gastric cancer who underwent R0 resection at Osaka City University Hospital (Osaka, Japan) between January 1997 and December 2012. All cancers were histopathologically confirmed as stage III gastric adenocarcinoma. Fourteen patients with concomitant malignancies, 13 patients who underwent neoadjuvant chemotherapy, 15 patients with incomplete preoperative laboratory data, and 3 patients who died of postoperative complications were excluded. Ultimately, this study included 225 patients. The Ethics Committee at our institution approved this retrospective study of clinical data study, which was conducted in according with the principles of the Declaration of Helsinki.

Blood samples were routinely obtained within 1 week before surgery. The CAR was calculated by dividing the serum C-reactive protein (CRP) value (mg/dL) by the serum albumin value (g/dL). The NLR was calculated as the number of neutrophils divided by the number of lymphocytes. The PLR was calculated as the number of platelets divided by the number of lymphocytes. The PNI was calculated as 10 × serum albumin value (g/dL) + 0.005 × lymphocytes (/mm^3^). The GPS was constructed from CRP and albumin values as follows. We allocated GPS of 2, 1, and 0 to patients with both elevated CRP (> 1.0 mg/dL) and hypoalbuminemia (< 3.5 g/dL), elevated CRP or hypoalbuminemia, and neither of these abnormalities, respectively. The PI constructed from the number of white blood cells (WBC) and CRP values are described as P2, P1, or P0 according to elevated CRP (> 1.0 mg/dL) and elevated WBC (< 11 × 10^9^/L), elevated CRP or elevated WBC, and neither of these abnormalities, respectively.

We evaluated the clinical variables of age, sex, body mass index (BMI), Eastern Cooperative Oncology Group (ECOG) performance status (PS), tumor location, macroscopic type, surgical procedure, lymph node dissection, histology, lymphatic invasion, venous invasion, TNM sub-stage, tumor size and adjuvant chemotherapy. Tumor stage was determined according to the third English edition of the Japanese classification of gastric carcinoma [16]. The median values served as the cut-off values for age, BMI, and tumor size. Time-dependent receiver operating characteristic (ROC) curves of 5-year OS as the endpoint were calculated to evaluate the predictive ability of CAR, NLR, PLR, and PNI, and maximal Youden indices were estimated to determine the cut-off values for these markers. All patients were classified as having high and low values according to these cut-off values.

Surgical procedures were determined according to tumor size, location, and the status of resection margins. In principle, adjuvant chemotherapy with oral fluoropyrimidines (5-FU, uracil-tegafur (UFT), 5’DFUR, or S-1) was administered to patients with good general condition who provided written, informed consent. The patients were followed every 4 months for the first 2 years, every 6 months for the next 3 years, and annually thereafter. Follow-up included physical examinations, routine blood tests, measurements of tumor markers, and enhanced abdominal CT scans. These were also implemented when recurrence was suspected. We contacted patients, family members, or their referring physicians to obtain appropriate follow-up data if patients had not presented for follow-up.

### Statistical analysis

Data were statistically analyzed using SPSS software (SPSS, Inc., Chicago, IL, USA), except for time-dependent ROC curves, which were analyzed using R-project Software, version 3.3.0. Overall survival and cancer-specific survival (CSS) rates were calculated from the date of the last surgical procedure until the date of the most recent follow-up or death, and to the date of most recent follow-up or death due to gastric cancer, respectively. These survival rates were calculated using the Kaplan-Meier method, and differences between curves were evaluated by log-rank tests Prognostic factors were investigated using Cox proportional hazards models, and hazard ratios (HRs) and 95% confidence intervals (CIs) were calculated. Separate multivariate analyses were performed to compare the prognostic values of individual inflammation-based and/or nutritional markers with *p* < 0.1 in univariate analyses because estimations of these variables overlapped. Values of *p* < 0.05 were considered to be statistically significant.

## Results

### Predictive ability and cut-off values of inflammation-based and/or nutritional markers

**Fig. 1:**
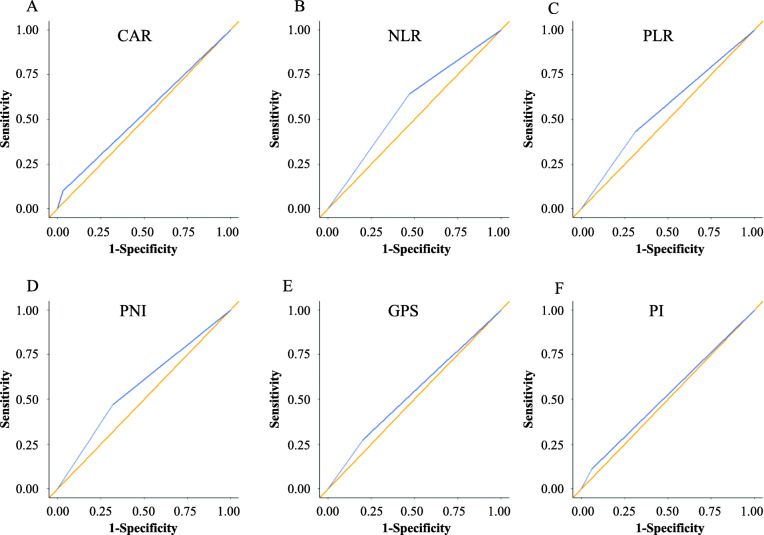
Time-dependent ROC curves of 5-year OS as the endpoint for CAR (**a**), NLR (**b**), PLR (**c**), GPS (**d**), PNI (**e**), and PI (**f**)

According to the results of time-dependent ROC analyses, we determined the cut-off values of 0.47 for CAR, 1.90 for NLR, 172 for PLR, 45.6 for PNI, 0 for GPS, and 0 for PI, respectively. When the patients were classified into two groups based on these cut-off values, the areas under the curve (AUC) for CAR, NLR, PLR, PNI, GPS, and PI were 0.534, 0.584, 0.558, 0.575, 0.536, and 0.524, respectively (Fig. 1). We classified 211 (93.8%) and 14 (6.2%) patients as having low and high CAR, respectively, 99 (44.0%) and 126 (56.0%) patients as having low and high NLR, respectively, 141 (62.7%) and 84 (37.3%) patients as having low and high PLR, respectively, and 90 (40.0%) and 135 (60.0%) patients as having low and high PNI, respectively.

### Patient demographics

Table 1 shows the relationships between clinicopathological characteristics and survival. The median age of the patients was 68 (interquartile range [IQR], 60–75) years, and 147 patients (75.3%) were male. The median BMI and tumor size were 21.9 (IQR 19.4–23.9) kg/m^2^ and 60.0 (IQR, 45.0–80.0) mm, respectively, and most patients had PS 0 (74.2%). The median CAR, NLR, PLR, and PNI were 0.031 (IQR, 0.023–0.115), 2.06 (IQR, 1.46–2.96), 153 (IQR, 111–208), and 46.8 (IQR, 42.5–49.9), respectively. Most patients had GPS 0 (76.0%) and PI 0 (91.1%).

**Table 1:** Univariate analyses of prognostic factors for OS of stage III gastric cancer

Variables	5-year OS (%)	No. of patients		Univariate	
		n	%	HR (95% CI)	*p* value
Total	48.7	225	100		
Age (years)					
≤ 68	54.0	117	52.0	1	
> 68	42.8	108	48.0	1.643 (1.164–2.319)	0.005
Sex					
Male	47.3	147	65.3	1	
Female	51.2	78	34.7	1.188 (0.823–1.715)	0.357
BMI (kg/m^2^)					
Low (≤ 21.9)	46.7	114	50.7	1	
High (> 21.9)	50.6	111	49.3	0.950 (0.674–1.339)	0.771
Performance status					
0	52.1	167	74.2	1	
1–3	39.1	58	25.8	1.606 (1.109–2.325)	0.012
Location					
Upper/Middle/Lower	50.7	209	92.9	1	
Whole	20.6	16	7.1	1.334 (1.092–1.630)	0.005
Macroscopic type					
Type 0/1/2	56.9	61	27.1	1	
Type 3/4/5	45.6	164	72.9	1.145 (0.774–1.693)	0.497
Operative procedure					
Distal gastrectomy	59.7	117	52.0	1	
Total gastrectomy	36.9	108	48.0	1.835 (1.295–2.600)	0.001
Lymph node dissection					
D1	49.8	32	14.2	1	
D2	48.6	193	85.8	0.911 (0.553–1.500)	0.713
Histology					
Differentiated	54.0	97	43.1	1	
Undifferentiated	44.4	128	56.9	1.198 (0.846–1.697)	0.308
Lymphatic invasion					
Absent	57.9	19	8.4	1	
Present	47.8	206	91.6	1.174 (0.633–2.177)	0.611
Venous invasion					
Absent	50.3	156	69.3	1	
Present	44.8	69	30.7	1.193 (0.830–1.716)	0.340
TNM sub-stage					
IIIA	62.2	80	35.6	1	0.011
IIIB	45.4	72	32.0	1.579 (1.030–2.420)	0.036
IIIC	36.2	73	32.4	1.908 (1.242–2.931)	0.003
Tumor size (mm)					
≤ 60	55.9	115	51.1	1	
> 60	40.9	110	48.9	1.534 (1.086–2.166)	0.015
Adjuvant chemotherapy					
Absent	40.1	41	18.2	1	
Present	50.6	184	81.8	0.575 (0.382–0.865)	0.008
CAR					
Low (≤ 0.47)	50.6	211	93.8	1	
High (> 0.47)	21.4	14	6.2	2.844 (1.561–5.181)	0.001
NLR					
Low (≤ 1.90)	58.0	99	44.0	1	
High (> 1.90)	41.2	126	56.0	1.400 (0.986–1.988)	0.060
PLR					
Low (≤ 172)	53.2	141	62.7	1	
High (> 172)	40.9	84	37.3	1.376 (0.970–1.953)	0.074
PNI					
Low (≤ 45.6)	39.0	90	40.0	1	
High (> 45.6)	54.9	135	60.0	0.730 (0.517–1.031)	0.074
GPS					
0	51.1	171	76.0	1	
1/2	41.1	54	24.0	1.463 (0.992–2.156)	0.055
PI					
0	50.1	205	91.1	1	
1/2	35.0	20	8.9	1.592 (0.914–2.773)	0.101

*BMI* body mass index, *PS* performance status, *TNM tumor-node-metastasis, CAR* C-reactive protein/Albumin ratio, *NLR neutrophil to lymphocyte ratio, PLR* platelet to lymphocyte ratio, *PNI* Prognostic Nutritional Index, *GPS Glasgow Prognostic Score, PI prognostic index*

### Survival

Survivors were followed for a median of 80 (IQR, 69–124) months. Seven patients were lost to follow-up, and the shortest follow-up period for survivors was 16 months. At the time of analysis, 131 (58.2%) patients had died. Disease recurred in 108 patients within a median duration of 14 (IQR, 7.7–26.1) months.

**Fig. 2:**
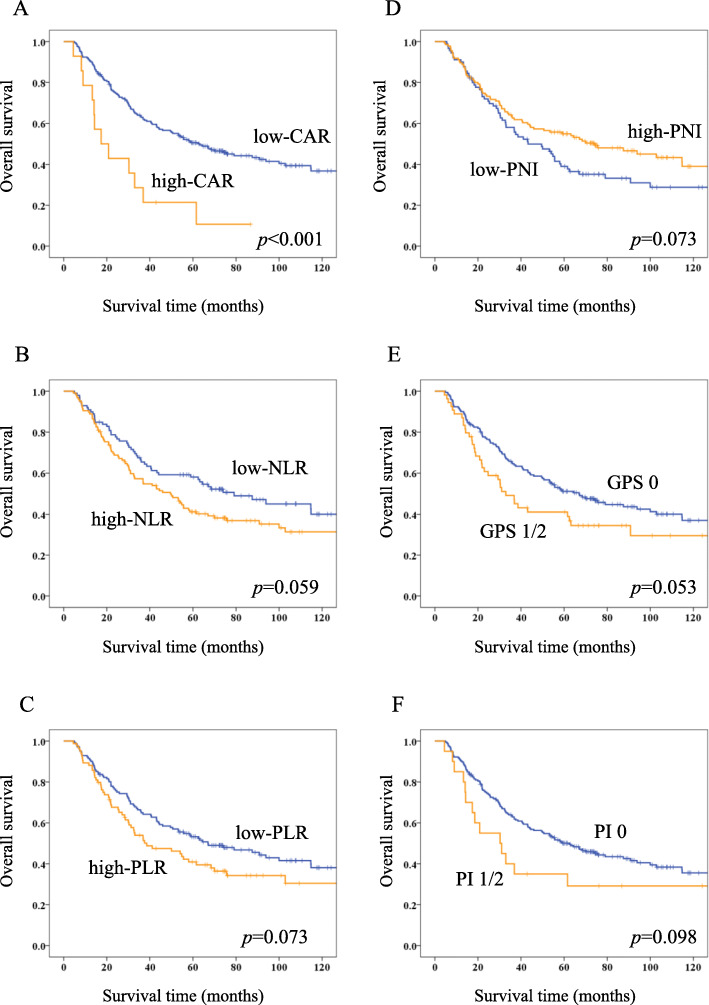
Kaplan-Meier survival curves of overall survival (OS) according to inflammation-based and/or nutritional markers Kaplan-Meier survival curves of OS according to CAR (**a**), NLR (**b**), PLR (**c**), GPS (**d**), PNI (**e**), and PI (**f**).

The 5-year OS rate for the entire study population was 48.7%. Figure 2a-f shows Kaplan-Meier survival curves for OS according to each inflammation-based and/or nutritional marker. The five-year OS rates in the groups with low and high CAR, NLR, PLR, PNI, GPS 0 and 1/2 groups, and PI 0 and 1/2 groups were 50.6 and 21.4% (*p* < 0.001), 58.0 and 41.2% (*p* = 0.059), 53.2 and 40.9% (*p* = 0.073), 39.0 and 54.9% (*p* = 0.073), 51.1 and 41.1% (*p* = 0.053), 50.1 and 35.0% (*p* = 0.098), respectively.Among these markers, OS significantly differed only between the two CAR groups.

### Prognostic factors for OS

Univariate analyses demonstrated that age, PS, location, operative procedure, TNM sub-stage, tumor size, adjuvant chemotherapy, and CAR were significantly associated with OS (Table 1). Multivariate analyses of each inflammation-based and/or nutritional marker with *p* < 0.1 in univariate analyses revealed that high CAR (HR, 2.257; 95% CI, 1.180–4.319; *p* = 0.014) and high-PLR (HR, 1.478; 95% CI, 1.025–2.133; *p* = 0.037) were independent predictors of worse OS (Table 2).

**Table 2:** Multivariate analyses of prognostic factors for OS of stage III gastric cancer

Variables	Analysis with CAR		Analysis with NLR		Analysis with PLR		Analysis with PNI		Analysis with GPS	
	HR (95% CI)	***p*** value	HR (95% CI)	***p*** value	HR (95% CI)	***p*** value	HR (95% CI)	***p*** value	HR (95% CI)	***p*** value
Age (years)										
≤ 68	1		1		1		1		1	
> 68	1.245 (0.832–1.862)	0.287	1.303 (0.878–1.935)	0.189	1.354 (0.910–2.014)	0.135	1.284 (0.851–1.937)	0.234	1.298 (0.871–1.934)	0.200
Performance status										
0	1		1		1		1		1	
1–3	1.246 (0.793–1.957)	0.340	1.311 (0.843–2.039)	0.229	1.320 (0.852–2.046)	0.214	1.333 (0.857–2.073)	0.202	1.318 (0.847–2.051)	0.221
Location										
Upper/Middle/Lower	1		1		1		1		1	
Whole	1.097 (0.882–1.364)	0.406	1.079 (0.866–1.345)	0.497	1.079 (0.867–1.343)	0.496	1.095 (0.880–1.362)	0.417	1.090 (0.876–1.358)	0.439
Operative procedure										
Distal gastrectomy	1		1		1		1		1	
Total gastrectomy	1.765 (1.201–2.595)	0.004	1.657 (1.138–2.413)	0.008	1.753 (1.195–2.573)	0.004	1.654 (1.132–2.417)	0.009	1.660 (1.136–2.425)	0.009
TNM sub-stage										
IIIA	1	0.060	1	0.048	1	0.034	1	0.066	1	0.063
IIIB	1.511 (0.972–2.348)	0.067	1.481 (0.953–2.300)	0.081	1.531 (0.983–2.382)	0.059	1.487 (0.953–2.321)	0.081	1.495 (0.962–2.323)	0.074
IIIC	1.694 (1.074–2.671)	0.023	1.754 (1.111–2.768)	0.016	1.812 (1.146–2.865)	0.011	1.707 (1.074–2.714)	0.024	1.702 (1.074–2.697)	0.024
Tumor size (mm)										
≤ 60	1		1		1		1		1	
> 60	1.017 (0.689–1.501)	0.933	1.065 (0.726–1.561)	0.749	0.983 (0.663–1.457)	0.931	1.058 (0.717–1.561)	0.776	1.054 (0.715–1.554)	0.791
Adjuvant chemotherapy										
Absent	1		1		1		1		1	
Present	0.807 (0.507–1.284)	0.366	0.810 (0.509–1.288)	0.373	0.772 (0.486–1.227)	0.274	0.797 (0.501–1.267)	0.337	0.806 (0.508–1.281)	0.361
CAR										
Low (≤0.47)	1									
High (> 0.47)	2.257 (1.180–4.319)	0.014								
NLR										
Low (≤1.90)			1							
High (> 1.90)			1.322 (0.926–1.887)	0.124						
PLR										
Low (≤172)					1					
High (> 172)					1.478 (1.025–2.133)	0.037				
PNI										
Low (≤45.6)							1			
High (> 45.6)							0.922 (0.631–1.348)	0.676		
GPS										
0									1	
1/2									1.141 (0.754–1.727)	0.533

*TNM tumor-node-metastasis, CAR* C-reactive protein/Albumin ratio, *NLR* neutrophil to lymphocyte ratio, PLR platelet to lymphocyte ratio, *PNI* Prognostic Nutritional Index, *GPS Glasgow* Prognostic Score

### Relationship between CAR, PLR and clinicopathological variables, and CSS

**Fig. 3:**
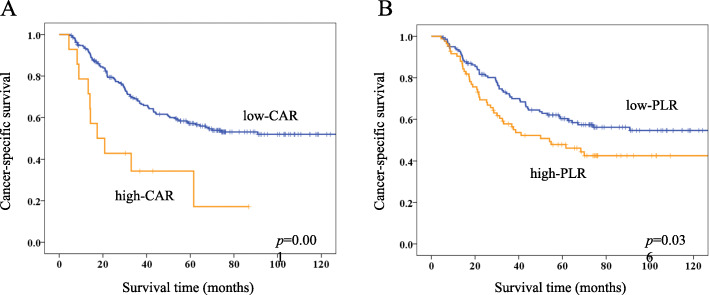
Kaplan-Meier survival curves of cancer-specific survival according to CAR (**a**) and PLR (**b**)

Table 3 summarizes the associations between clinicopathological variables and CAR and PLR. CAR was significantly associated with age (*p* = 0.004), PS (*p* = 0.006), venous invasion (0.027), tumor size (*p* = 0.027), and all other inflammation-based and/or nutritional markers (NLR, *p* = 0.004; PLR, PNI, GPS and PI, *p* < 0.001). The PLR was significantly associated with sex (*p* = 0.046), BMI (*p* = 0.004), tumor size (*p* = 0.001), and all other inflammation-based and/or nutritional markers (GPS, *p* = 0.011; PI, *p* = 0.002; CAR, NLR and PNI, *p* < 0.001). Figure 3a and b shows Kaplan-Meier survival curves for CSS according to CAR and PLR. The 5-year CSS rates in the groups with low and high CAR were 57.2 and 34.3% (*p* = 0.001), respectively, and 60.3 and 47.8% (*p* = 0.036) in those with low and high PLR, respectively. Multivariate analyses of clinical variables with *p* < 0.1 in univariate analyses and CAR and PLR revealed that high CAR (HR, 2.771; 95% CI, 1.398–5.493; *p* = 0.004) and high PLR (HR, 1.552; 95% CI, 1.029–2.341; *p* = 0.036) were independent prognostic factors for CSS (Table 4).

**Table 3:** Correlations between CAR, PLR and clinicopathological characteristics of patients

Variables	CAR				*p* value	PLR				*p* value
	Low		High			Low		High		
	n	%	n	%		n	%	n	%	
Age (years)										
≤ 68	115	54.5	2	14.3		74	52.5	43	51.2	
> 68	96	45.5	12	85.7	0.004	67	47.5	41	48.8	0.851
Sex										
Male	136	64.5	11	78.6		99	70.2	48	57.1	
Female	75	35.5	3	21.4	0.389	42	29.8	36	42.9	0.046
BMI (kg/m^2^)										
Low (≤21.9)	105	49.8	9	64.3		61	43.3	53	63.1	
High (> 21.9)	106	50.2	5	35.7	0.293	80	56.7	31	36.9	0.004
Performance status										
0	161	76.3	6	42.9		107	75.9	60	71.4	
1–3	50	23.7	8	57.1	0.006	34	24.1	24	28.6	0.460
Location										
U/M/L	197	93.4	12	85.7		132	93.6	77	91.7	
Whole	14	6.6	2	14.3	0.261	9	6.4	7	8.3	0.582
Macroscopic type										
Type 0/1/2	59	28.0	2	14.3		41	29.1	20	23.8	
Type 3/4/5	152	72.0	12	85.7	0.361	100	70.9	64	76.2	0.390
Operative procedure										
Distal gastrectomy	110	52.1	7	50.0		72	51.1	45	53.6	
Total gastrectomy	101	47.9	7	50.0	0.877	69	48.9	39	46.4	0.716
Lymph node dissection										
D1	30	14.2	2	14.3		19	13.5	13	15.5	
D2	181	85.8	12	85.7	1.000	122	86.5	71	84.5	0.678
Histology										
Differentiated	91	43.1	6	42.9		58	41.1	39	46.4	
Undifferentiated	120	56.9	8	57.1	0.984	83	58.9	45	53.6	0.438
Lymphatic invasion										
Absent	18	8.5	1	7.1		13	9.2	6	7.1	
Present	193	91.5	13	92.9	1.000	128	90.8	78	92.9	0.588
Venous invasion										
Absent	150	71.1	6	42.9		102	72.3	54	64.3	
Present	61	28.9	8	57.1	0.027	39	27.7	30	35.7	0.205
TNM sub-stage										
IIIA	77	36.5	3	21.4		54	38.3	26	31.0	
IIIB	69	32.7	3	21.4		41	29.1	31	36.9	
IIIC	55	26.1	8	57.1	0.125	46	32.6	27	32.1	0.404
Tumor size (mm)										
≤ 60	112	53.1	3	21.4		84	59.6	31	36.9	
> 60	99	46.9	11	78.6	0.027	57	40.4	53	63.1	0.001
Adjuvant chemotherapy										
Absent	37	17.5	4	28.6		29	20.6	12	14.3	
Present	174	82.5	10	71.4	0.292	112	79.4	72	85.7	0.238
CAR										
Low (≤0.47)						139	98.6	72	85.7	
High (> 0.47)						2	1.4	12	14.3	< 0.001
NLR										
Low (≤1.90)	98	46.4	1	7.1		85	60.3	14	16.7	
High (> 1.90)	113	53.6	13	92.9	0.004	56	39.7	70	83.3	< 0.001
PLR										
Low (≤172)	139	65.9	2	85.7						
High (> 172)	72	34.1	12	85.7	< 0.001					
PNI										
Low (≤45.6)	78	37.0	12	85.7		37	26.2	53	63.1	
High (> 45.6)	133	63.0	2	14.3	< 0.001	104	73.8	31	36.9	< 0.001
GPS										
0	171	81.0	0	0		115	81.6	56	66.7	
1/2	40	19.0	14	100	< 0.001	26	18.4	28	33.3	0.011
PI										
0	205	97.2	0	0		135	95.7	70	83.3	
1/2	6	2.8	14	100	< 0.001	6	4.3	14	16.7	0.002
Recurrence										
Absent	113	53.6	4	28.6		80	56.7	37	44.0	
Present	98	46.4	10	71.4	0.097	61	43.3	47	56.0	0.065

*BMI* body mass index, *PS* performance status, TNM tumor-node-metastasis, *CAR* C-reactive protein/Albumin ratio, *NLR* neutrophil to lymphocyte ratio, *PLR* platelet to lymphocyte ratio, *PNI* Prognostic Nutritional Index, *GPS Glasgow Prognostic Score, PI prognostic index*

**Table 4:** Multivariate analyses of prognostic factors for CSS of stage III gastric cancer

Variables	Analysis with CAR		Analysis with PLR	
	HR (95% CI)	***p*** value	HR (95% CI)	***p*** value
Location				
Upper/Middle/Lower	1		1	
Whole	1.099 (0865–1.395)	0.439	1.083 (0.852–1.376)	0.516
Operative procedure				
Distal gastrectomy	1		1	
Total gastrectomy	1.723 (1.110–2.676)	0.015	1.705 (1.100–2.643)	0.017
TNM sub-stage				
IIIA	1	0.002	1	0.001
IIIB	2.019 (1.187–3.435)	0.010	2.015 (1.184–3.432)	0.010
IIIC	2.617 (1.534–4.467)	< 0.001	2.799 (1.635–4.792)	< 0.001
Tumor size (mm)				
≤ 60	1		1	
> 60	0.978 (0.630–1.519)	0.921	0.946 (0.605–1.481)	0.809
CAR				
Low (≤0.47)	1			
High (> 0.47)	2.771 (1.398–5.493)	0.004		
PLR				
Low (≤172)			1	
High (> 172)			1.552 (1.029–2.341)	0.036

*TNM tumor-node-metastasis, CAR* C-reactive protein/Albumin ratio, *PLR* platelet to lymphocyte ratio

### Combined index

**Fig. 4:**
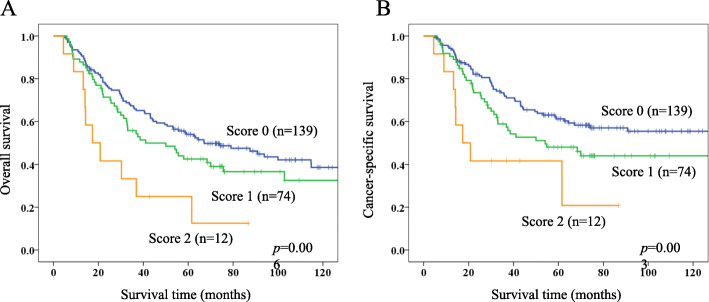
Kaplan-Meier survival curves of overall survival (**a**) and cancer-specific survival (**b**) according to CAR-PLR score

According to the results of multivariate analyses, we constructed CAR-PLR score as a prognostic index, as follows: CAR-PLR score 2, both high-CAR and high-PLR; CAR-PLR score 1, either high-CAR or high-PLR, but not both; and CAR-PLR score 0, neither abnormality. CAR-PLR scores were 0 for 139 patients (61.8%), 1 for 74 patients (32.9%), and 2 for 12 patients (5.3%). The AUC of CAR-PLR score for predicting 5-year OS was 0.573. Kaplan-Meier survival curves for OS and CSS according to CAR-PLR score are shown in Fig. 4a, b. Five-year OS and CSS rates for the CAR-PLR score 0, 1, and 2 groups were 54.0, 42.6, and 25.0%(*p* = 0.006), and 61.2, 48.0, and 41.7% (*p* = 0.003), respectively.

## Discussion

The present study evaluated and compared the prognostic values of preoperative CAR, NLR, PLR, PNI, GPS, and PI in patients with stage III gastric cancer after curative resection and verified that the results of the same analyses differed between stages II and III. We found that CAR and PLR with cut-off values of 0.47 and 172, respectively, were independent prognostic factors for OS and CSS in patients with stage III gastric cancer. These results were quite different from our previous findings in patients with stage II gastric cancer who were analyzed in the same manner [15]. To the best of our knowledge, this is the first investigation of differences in optimal inflammation-based and/or nutritional markers and optimal cut-off values as prognostic factors between stage II and III gastric cancer.

The mechanisms underlying the relationship between systemic inflammation and/or nutritional status and the prognosis of cancer patients are complex and not yet fully elucidated. The PLR has been investigated in detail as a prognostic inflammation-based marker, and it is a prognostic factor for various types of cancer [8, 17]. Several studies did not find that PLR is a prognostic factor for gastric cancer [18, 19]; however, a recent meta-analysis revealed that PLR does have prognostic value for gastric cancer [20]. We identified PLR as an independent prognostic factor for patients with stage III, but not stage II cancer [15]. A higher PLR indicates relatively more platelets and fewer lymphocytes. Platelets are involved in tumor progression and metastasis. Some growth factors secreted from platelets in the tumor microenvironment, such as transforming growth factor beta (TGF-β), vascular endothelial growth factor (VEGF) and platelet derived growth factor (PDGF) contribute to tumor growth, angiogenesis, and tumoral neovascularization [21]. Lymphocytes play a prominent role in antitumor immunity, and lymphopenia has been associated with a worse prognosis in various cancers [22, 23]. Thus, a high PLR might have negatively affected tumor progression, which could have contributed to the poorer OS and CSS observed in the present study. Furthermore, micrometastases and residual cancer cells might be more prevalent in stage III than in stage II cancers after R0 resection. This could also explain why PLR was a prognostic factor for stage III, but not stage II gastric cancer. Consistent with our findings, a meta-analysis of 14 cohorts by Gu et al. concluded that elevated PLR indicated poor OS in patients with advanced, but not early stage gastric cancer [20].

We identified CAR as an independent prognostic factor in patients with stage III, as well as stage II gastric cancer, although the AUC and cutoffs were quite different for each stage. Tumor progression causes systemic inflammation and impaired nutritional status in patients with cancer. CRP is an established acute reactive protein synthesized by hepatocytes under conditions of systemic inflammation. Serum albumin is commonly used as an indicator of nutritional status. Furthermore, either serum CRP or albumin level alone are independent prognostic factors in several malignancies [24–27]. Therefore, a higher CAR might reflect a better systemic inflammatory response and worse nutritional status among cancer patients due to tumor progression and might serve as a prognostic indicator. Indeed, CAR has been indicated as an independent prognostic factor in various malignancies, including gastric cancer [3, 4]. Liu et al. reported that among NLR, PLR, GPS, and CAR, only CAR with a cut-off value of 0.025 was an independent prognostic factor in patients with gastric cancer undergoing curative resection, and a subgroup analysis according to tumor stage revealed that CAR with a cut-off value of 0.025 was significantly associated with OS in stages II and III [19]. Although CAR with a cut-off value of 0.47 was an independent prognostic factor among patients with stage III gastric cancer in the present study, the AUC of CAR with 0.510 in stage III gastric cancer was considerably lower than that of 0.641 in stage II. This finding suggests that the influence of preoperative patient-related factors represented by CAR on prognosis weakens at the more advanced stages of cancer.

We recently reported the prognostic impact of the same preoperative inflammation-based and/or nutritional markers analyzed herein in patients with stage II gastric cancer [15]. We speculated that to minimize inconsistency of results among studies, the optimal value of preoperative inflammation-based and/or nutritional markers and their cut-off values should be determined based on a specific population with a similar prognosis, which might help to make such markers reliable enough for use in clinical practice. As the appropriate method to determine cut-off values for inflammation-based and/or nutritional markers has not been established, we determined cut-off values in the present study from time-dependent ROC curve analyses, which seemed to confer advantages in terms of objectivity. Furthermore, to verify our speculation, we applied the same analyses to stage II and stage III gastric cancer. Consequently, CAR and PNI with cut-off values of 0.03 and 49.2, respectively, were independent prognostic factors for OS in stage II gastric cancer, whereas CAR with a cut-off value of 0.47 and PLR with a cut-off value of 172 were independent prognostic factors in stage III. These findings supported our hypothesis that the optimal marker and cut-off values should be determined based on a specific population. On the other hand, our findings were inconsistent with those of Wang et al. [28] who found that among PNI, NLR, the lymphocyte to monocyte ratio, and PLR, only PNI was an independent prognostic factor in patients with stage III gastric cancer after curative resection, which was similar population to ours. They used median values as cut-off values for inflammation-based and/or nutritional markers, and a different method of multivariate analysis from the present study. Therefore, to reduce discrepancies among the results of various studies, a reliable and uniform method should be established to determine optimal inflammation-based and/or nutritional markers and their cut-off values to predict the prognosis of patients with cancer.

This retrospective study at a single institution has several potential limitations. The study design might have some correlation with selection bias. Our study population was relatively small because only pathological stage III gastric cancer was the study focus. Some potential cofactors affecting inflammation-based and/or nutritional markers were not controlled. Further large-scale prospective validation studies are needed to confirm our findings.

## Conclusions

We found that CAR and PLR are independent prognostic factors for OS and CSS. These markers show more promise in terms of predicting prognosis than the established inflammation-based and/or nutritional markers, NLR, PNI, GPS, and PI in patients with stage III gastric cancer after curative resection. The optimal inflammation-based and/or nutritional markers and their cut-off values for predicting the prognosis of patients with cancer should be determined in specific populations so that they can become applicable to daily clinical practice.

## Data Availability

The datasets used and/or analyzed during the current study are available from the corresponding author on reasonable request.

## Abbreviations

OS: Overall survival
CAR: C-reactive protein to albumin ratio
NLR: Neutrophils to lymphocyte ratio
PLR: Platelets to lymphocyte ratio
PNI: Prognostic nutritional index
GPS: Glasgow prognostic score
PI: Prognostic index
CRP: C-reactive protein
BMI: Body mass index
ECOG: Eastern Cooperative Oncology Group
PS: Performance status
ROC: Receiver operating characteristic
UFT: Uracil-tegafur
CT: Computed tomography
CSS: Cancer-specific survival
HR: Hazard ratio
CI: Confidence interval
AUC: Areas under the curve
IQR: Interquartile range
TGF-β: Transforming growth factor beta
VEGF: Vascular endothelial growth factor
PDGF: Platelet derived growth factor
